# Small Bowel Obstruction due to Meckel's Diverticulum: A Case Report

**DOI:** 10.1002/ccr3.71643

**Published:** 2025-12-10

**Authors:** Mansab Ali, Amir Usman, Shahr Yar, Bilal Aslam, Salman Ghulam Nabi, Seemab Ara, Dawood Azam Farooq, Aiman Tanveer, Sheraz Arshad, Nour Fakih, Muhammad Imran

**Affiliations:** ^1^ Department of General Surgery University College of Medicine and Dentistry, The University of Lahore Lahore Pakistan; ^2^ Department of General Surgery Glangwili General Hospital Carmarthen UK; ^3^ Faculty of Medicine University College of Medicine and Dentistry, The University of Lahore Lahore Pakistan; ^4^ Department of Natural Sciences Lebanese American University Beirut Lebanon

## Abstract

Meckel's diverticulum should be considered in cases of unexplained small bowel obstruction, especially in pediatric and adolescent patients. Early recognition and timely surgical intervention can prevent complications and lead to favorable outcomes.

**Abbreviations**
CT: computed tomography
DRE: digital rectal examination
GI: gastrointestinal
Hb: hemoglobin
NPO: nothing by mouth
RIF: right iliac fossa
RR: respiratory rate
SpO_2_
: oxygen saturation
WBCs: white blood cells

## 1 Introduction

Meckel's diverticulum (MD) is the most common congenital anomaly of the gastrointestinal (GI) tract, occurring in approximately 2% of the population [1]. It results from the incomplete obliteration of the omphalomesenteric duct during fetal development and is classified as a true diverticulum containing all three layers of the intestinal wall [2]. Although most cases remain asymptomatic, complications such as gastrointestinal bleeding, inflammation, perforation, and small bowel obstruction (SBO) can occur, affecting about 2% of MD cases [3, 4].

Small bowel obstruction due to MD is rare but can arise from fibrous bands, volvulus, intussusception, Littre's hernia, or inflammatory adhesions [5]. Among these, fibrous bands tethering the diverticulum to adjacent structures can create an extrinsic obstruction, necessitating prompt surgical intervention [6].

This case report presents a 14‐year‐old male with small bowel obstruction due to a fibrous band associated with MD. The case underscores the importance of considering MD in pediatric and adolescent patients presenting with intestinal obstruction and highlights the role of early surgical intervention in preventing complications.

## 2 Case Presentation/Examination

### 2.1 Case History

A 14‐year‐old male with a history of appendectomy presented to the emergency department of the University of Lahore Teaching Hospital with pain in the right iliac fossa (RIF), associated with fever and constipation for the past 3 days. However, the patient denied any diarrhea, vomiting, or rectal bleeding. There was also no history of dysuria or lower urinary tract symptoms.

On examination, the patient was febrile (101°F), with a pulse rate of 92/min, blood pressure of 110/64 mmHg, respiratory rate of 19/min, and SpO_2_ of 98% on room air. The abdomen was soft and mildly distended, with tenderness localized to the RIF but no rebound tenderness. Bowel sounds were hyperactive, and shifting dullness and fluid thrill were negative. Digital rectal examination (DRE) revealed an empty rectum. No palpable masses or visceromegaly were noted.

The patient was admitted for further evaluation and kept nil per os (NPO). He was started on intravenous fluids, antibiotics, and analgesics. Urinary catheterization was performed for urine output monitoring. A nasogastric tube was inserted for decompression.

## 3 Methods

### 3.1 Laboratory Investigations

- WBCs: 9 × 10^9^
- Hb: 13 g/dL
- Platelets: 465,000
- Liver function tests, renal function tests, urinalysis, and electrolytes were within normal limits.

### 3.2 Differential Diagnosis

Given the patient's clinical presentation of SBO without prior similar episodes, several potential etiologies were considered. The differential diagnosis included intussusception, adhesions, volvulus, hernias (e.g., Littre's hernia), and MD.

- Meckel’s diverticulum (MD): MD was strongly suspected based on imaging findings. Given its potential to cause SBO via fibrous bands, intussusception, or volvulus, further diagnostic steps were warranted.
- *Intussusception*: In adults, intussusception is often associated with a pathological lead point, such as a polyp, tumor, or MD. The patient's symptoms and imaging findings raised concern for this possibility, prompting further evaluation.
- *Adhesions*: Post‐surgical adhesions are the most frequent cause of SBO. However, given the absence of prior abdominal surgery or intra‐abdominal inflammation, adhesive disease was considered less likely.
- *Volvulus*: Although volvulus can present with similar obstructive symptoms, imaging did not demonstrate the characteristic “whirlpool sign” or evidence of sigmoid/cecal twisting.
- *Hernias (e.g., Littre's hernia)*: A Littre's hernia involves the protrusion of MD through a hernia defect. While external hernias can contribute to SBO, physical examination and imaging showed no evidence of an external hernia or strangulation.

### 3.3 Imaging and Diagnosis

An abdominal X‐ray in both erect and supine positions showed dilated small bowel loops with air‐fluid levels but no air under the diaphragm. An abdominal ultrasound also showed dilated bowel loops with sluggish peristalsis, but no bowel wall edema, mass, or hernia.

Given the suspicion of MD or intussusception, diagnostic laparoscopy was performed by a general surgeon. It revealed a fibrous band originating from MD, causing obstruction at the level of the terminal ileum. The proximal small bowel was dilated, while the distal loops were collapsed.

### 3.4 Surgical Management

**FIGURE 1:**
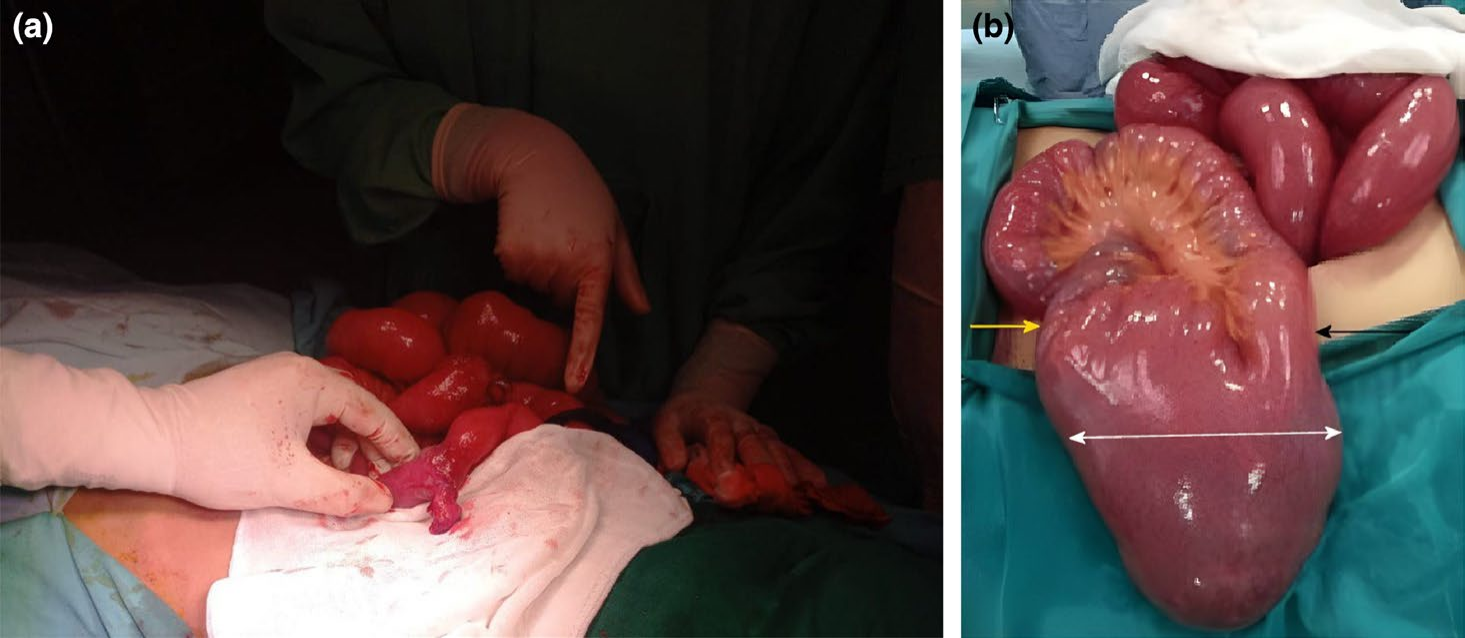
(a) Giant Meckel's diverticulum causing gastrointestinal obstruction. The proximal dilated small bowel loops observed indicate an SBO. (b) White arrow: diameter of Meckel's diverticulum; Black and Yellow arrows indicate the proximal and distil segments of Meckel's diverticulum.

An exploratory laparotomy was performed, revealing a fibrous band originating from MD, causing obstruction at the level of the terminal ileum. MD is shown in Figure 1. The band was divided, and the diverticulum was excised. No intraoperative complications were encountered, and the bowel was viable.

## 4 Conclusion and Results

This case illustrates a rare but significant presentation of small bowel obstruction caused by a fibrous band attached to Meckel's diverticulum. The patient had an uneventful recovery and remained symptom‐free at the four‐month follow‐up, reflecting the excellent prognosis typically seen after surgical resection of symptomatic MD. In cases of SBO, early surgical intervention is crucial to avoid serious complications such as bowel ischemia or perforation.

## 5 Discussion

This case highlights the diagnostic and therapeutic challenges associated with MD as a rare cause of SBO in pediatric patients. Although MD is the most common congenital anomaly of the GI tract, complications such as obstruction due to a fibrous band are uncommon and can present diagnostic difficulties, particularly in adolescents [7]. The incidence of MD‐related obstruction varies widely, but studies estimate that it accounts for approximately 2% of all MD complications, emphasizing its rarity [8]. This case underscores the importance of maintaining a high index of suspicion for MD in pediatric and adolescent patients with atypical presentations of intestinal obstruction and highlights the limitations of routine imaging in diagnosing MD preoperatively.

The patient, a 14‐year‐old male, presented with fever and constipation. Clinical examination revealed RIF tenderness, a mildly distended abdomen with hyperactive bowel sounds, but no palpable masses or signs of peritonitis. Although vomiting and abdominal distension are typical features of SBO [9], these symptoms were not prominent in this case. Imaging studies, including abdominal X‐rays and ultrasound, demonstrated dilated small bowel loops with sluggish peristalsis, raising suspicion of SBO. This presentation aligns with previous reports of MD‐associated fibrous bands causing small bowel obstruction, which, though rare, represents a clinically significant complication [10]. Given the patient's clinical presentation and imaging findings, the differential diagnosis included intussusception, adhesions, volvulus, hernias (e.g., Littre's hernia), and MD. However, the absence of rectal bleeding, a palpable abdominal mass, or prior history of abdominal surgery helped narrow the differential, making MD with a fibrous band the most probable etiology [11, 12].

The preoperative diagnosis of MD remains challenging due to its nonspecific clinical presentation and imaging limitations. While X‐rays and ultrasound can suggest SBO, they are not definitive for MD [2, 13]. In this case, imaging showed dilated small bowel loops with sluggish peristalsis, indicating obstruction but failing to localize the exact cause. The Meckel radionuclide scan, the most sensitive test for detecting MD with ectopic gastric mucosa, has limited utility in cases where the complication is mechanical obstruction rather than gastrointestinal bleeding [13]. Instead, diagnostic laparoscopy played a pivotal role, enabling both confirmation of the diagnosis and immediate surgical intervention. Laparoscopy is increasingly recognized as a valuable diagnostic and therapeutic tool for MD‐related complications, offering a minimally invasive approach with direct visualization of the pathology [14].

Surgical resection remains the gold standard treatment for symptomatic MD, particularly in cases involving intestinal obstruction, hemorrhage, or perforation. In this patient, an exploratory laparotomy was performed, the fibrous band was divided, and the diverticulum was excised. The procedure was conducted by a general surgeon, adhering to standard surgical protocols. This approach aligns with current guidelines for managing symptomatic MD and is crucial for preventing complications such as ischemia, perforation, or recurrent obstruction [15]. The patient's uneventful recovery and symptom‐free status at 4 months reinforce the excellent prognosis typically associated with timely surgical intervention for symptomatic MD [6].

In addition to symptomatic cases, incidental findings of MD during unrelated abdominal surgeries are common. Whether such asymptomatic diverticula should be resected remains a subject of debate. Current literature, including a systematic review by Yagnik et al. [16], provides insights into this dilemma. The review highlights that incidental MD with risk factors such as a diverticulum larger than 2 cm, the presence of ectopic mucosa, or associated fibrous bands increases the likelihood of symptomatic presentations and might benefit from prophylactic resection. However, in the absence of such risk factors, routine resection may not be warranted due to the potential for surgical complications. Addressing these considerations provides a more comprehensive understanding of the clinical approach to both symptomatic and incidental MD.

This case contributes to the growing body of evidence emphasizing the need to consider MD in the differential diagnosis of pediatric SBO, particularly when associated with a fibrous band. Early recognition and timely surgical management are critical for preventing complications and ensuring optimal patient outcomes. Increased clinical awareness and judicious use of diagnostic laparoscopy can facilitate accurate diagnosis and effective intervention, ultimately improving the prognosis of affected patients. Future research should focus on refining indications for prophylactic resection of incidentally discovered MD and optimizing minimally invasive techniques to improve patient outcomes.

## Author Contributions

**Mansab Ali:** validation, visualization, writing – original draft, writing – review and editing. **Amir Usman:** writing – original draft, writing – review and editing. **Shahr Yar:** writing – original draft, writing – review and editing. **Bilal Aslam:** writing – original draft, writing – review and editing. **Salman Ghulam Nabi:** writing – original draft, writing – review and editing. **Seemab Ara:** validation. **Dawood Azam Farooq:** writing – original draft, writing – review and editing. **Aiman Tanveer:** writing – original draft, writing – review and editing. **Sheraz Arshad:** writing – original draft, writing – review and editing. **Nour Fakih:** writing – original draft, writing – review and editing. **Muhammad Imran:** supervision, validation, writing – review and editing.

## Funding

The authors have nothing to report.

## Ethics Statement

The publication of this case report has been authorized by the quality service of our institution because case reports are exempt from ethical approval in our institute.

## Consent

Written informed consent was obtained from the individual for the publication of any potentially identifiable images or data included in this article.

## Conflicts of Interest

The authors declare no conflicts of interest.

## Data Availability

Due to privacy and ethical restrictions, the data supporting the findings of this study are available from the corresponding author only upon reasonable request.
